# Case Report: Immune checkpoint inhibitor-induced IgG4-related disease mimicking renal metastatic progression: successful steroid-sparing management with rituximab

**DOI:** 10.3389/fimmu.2026.1884821

**Published:** 2026-07-15

**Authors:** Mayara Elisa Bonatto, Jan Dvořák, Marek Kollár, David Girsa, Miroslav Průcha, Šárka Forejtová, Heřman Mann, Jiří Vencovský, Karel Pavelka, Ladislav Šenolt

**Affiliations:** 1 Institute of Rheumatology and Department of Rheumatology, 1st Faculty of Medicine, Charles University, Prague, Czechia; 2 Department of Oncology, Faculty Hospital Královské Vinohrady, Prague, Czechia; 3 Department of Pathology, Institute for Clinical and Experimental Medicine, Prague, Czechia; 4 Department of Radiology, Faculty Hospital Královské Vinohrady, Prague, Czechia; 5 Department of Allergology and Clinical Immunology, Hospital na Homolce, Prague, Czechia

**Keywords:** cancer immunotherapy, IgG4-related disease, immune checkpoint inhibitors, immune-related adverse events, ipilimumab, nivolumab, renal pseudotumor, rituximab

## Abstract

**Background:**

Immune checkpoint inhibitors (ICIs) can induce a broad spectrum of immune-related adverse events (irAEs), including rare fibroinflammatory autoimmune manifestations. IgG4-related disease (IgG4-RD) has only exceptionally been described following dual ICI therapy.

**Case presentation:**

We report a 65-year-old man with metastatic clear cell renal carcinoma treated with nivolumab plus ipilimumab after nephrectomy. Despite initial radiologic stability, follow-up computed tomography revealed newly developed mass-like lesions in the solitary remaining kidney, raising suspicion of metastatic progression. CT-guided biopsy demonstrated subacute pyelonephritis, acute tubular injury, storiform fibrosis, and dense IgG4-positive plasma cell infiltration consistent with IgG4-RD. Because glucocorticoids were considered potentially detrimental to antitumor immune surveillance, rituximab was selected as steroid-sparing first-line therapy. Two infusions of rituximab (1000 mg each) led to radiologic stabilization/regression of renal lesions while pulmonary metastases remained under oncologic control.

**Conclusion:**

This case highlights IgG4-RD as a rare but clinically important underrecognized irAE of dual ICI therapy that may mimic malignant progression. Histologic confirmation is crucial, and B-cell depletion with rituximab may represent an effective treatment strategy when preservation of antitumor immunity is a priority.

## Introduction

Immune checkpoint inhibitors (ICIs) have fundamentally changed the therapeutic landscape of advanced malignancies by restoring antitumor T-cell activity and inducing durable clinical responses (1). Combined blockade of programmed cell death protein 1 (PD-1) and cytotoxic T-lymphocyte-associated antigen 4 (CTLA-4), represented by nivolumab plus ipilimumab, provides superior oncologic efficacy in several cancers but is also associated with a substantial burden of immune-related adverse events (irAEs) resulting from disruption of peripheral immune tolerance (2, 3).

Although dermatologic, gastrointestinal, hepatic, and endocrine toxicities are the most common manifestations, rheumatic and other systemic immune-mediated complications are increasingly recognized (4–6). Among these, IgG4-related disease (IgG4-RD) appears to be exceptionally rare.

IgG4-RD is a chronic immune-mediated fibroinflammatory condition characterized by tumefactive organ enlargement, storiform fibrosis, obliterative phlebitis, and dense infiltration of IgG4-positive plasma cells. The disease can affect nearly every organ system, including pancreas, kidneys, lungs, retroperitoneum, bile ducts, salivary glands, and lymph nodes (7, 8, 11, 12). Owing to its indolent course and mass-forming radiologic appearance, IgG4-RD frequently mimics malignant, infectious, or infiltrative disorders.

Only isolated cases of ICI-triggered IgG4-RD have been described to date (9, 10). We report a histologically confirmed case of renal IgG4-RD arising after nivolumab plus ipilimumab therapy for metastatic clear cell renal carcinoma and discuss its possible immunopathogenic basis and therapeutic implications.

## Case presentation

A 65-year-old man was diagnosed with clear cell renal carcinoma (T3aNXM1, G3) of the right kidney (**Figure 1A**), with pulmonary metastatic lesions identified on baseline staging (**Figure 1B**). He underwent right nephrectomy followed by four cycles of combined nivolumab and ipilimumab administered between October and December 2023.

**Figure 1:**
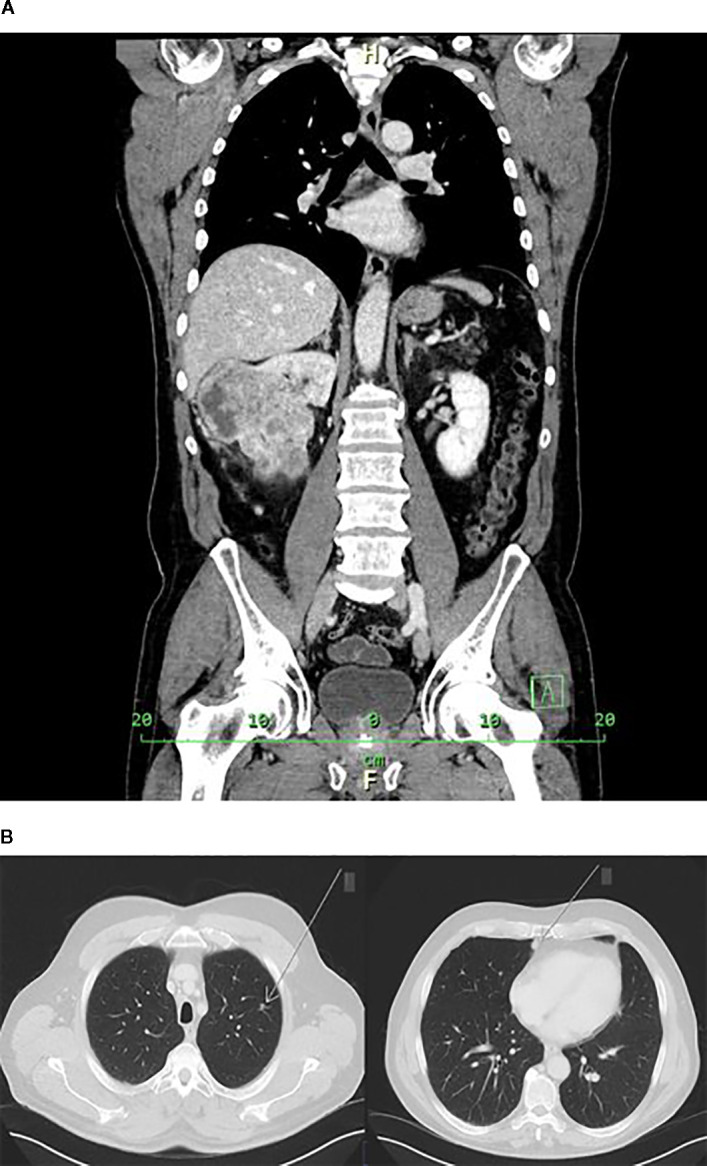
**(A)** Initial imaging in the frontal section showing clear cell renal carcinoma of the right kidney. **(B)** Metastatic lesions in the lungs (arrows).

Restaging computed tomography (CT) in January 2024 demonstrated stable oncologic disease without progression. Clinically, the patient remained asymptomatic, reporting no pain, constitutional symptoms, or functional limitations. The patient was not on any concomitant medication other than the prescribed anticancer therapy. (A) Laboratory evaluation showed only mild renal dysfunction (creatinine 130 µmol/L), low-grade proteinuria (protein-to-creatinine ratio 22 mg/mmol), and mildly elevated C-reactive protein (5.5 mg/L). Immunological evaluation revealed negative antinuclear antibodies (ANA), extractable nuclear antigen antibodies (ENA) including anti-Ro52 a anti-Ro60, anti-dsDNA antibodies, and anti-neutrophil cytoplasmic antibodies (anti-MPO and anti-PR3). Complement levels (C3 and C4) were within the normal range, and serum IgG and IgG4 concentrations were not elevated. (B).

However, repeat CT imaging performed in March 2024 revealed newly developed mass-like lesions within the solitary remaining kidney (**Figure 2**), raising suspicion of metastatic recurrence. Because of the indeterminate radiologic appearance, CT-guided renal biopsy was undertaken.

**Figure 2:**
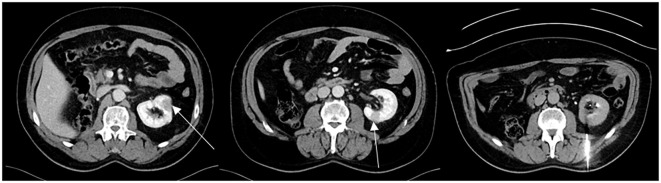
Newly detected renal lesions (arrows) and CT-guided biopsy of renal lesion following immune checkpoint inhibitor therapy. .

Histopathological examination demonstrated subacute pyelonephritis and acute tubular injury accompanied by marked fibroinflammatory changes. Dense lymphoplasmacytic infiltrates rich in IgG4-positive plasma cells together with storiform fibrosis strongly supported the diagnosis of IgG4-related disease (**Figure 3**). Granulomas were not identified in the renal biopsy specimen. Their absence argues against granulomatous tubulointerstitial nephritis and is also not supportive of alternative granulomatous inflammatory conditions. In contrast, the biopsy demonstrated characteristic storiform fibrosis, which represents one of the major histopathological features of IgG4-related disease and is generally not observed in drug-induced tubulointerstitial nephritis or granulomatous tubulous interstitial nephritis. Although storiform fibrosis may rarely be encountered in other conditions such as malakoplakia, the overall clinicopathological context was not consistent with this diagnosis. However, the histopathological findings did not suggest membranous nephropathy. Specifically, glomeruli did not show the characteristic morphological features of membranous nephropathy, such as diffuse thickening of the glomerular basement membranes. Taken together, the presence of storiform fibrosis and the absence of histological features supporting alternative diagnoses were considered consistent with IgG4-related tubulointerstitial nephritis in the appropriate clinical setting (C).

**Figure 3:**
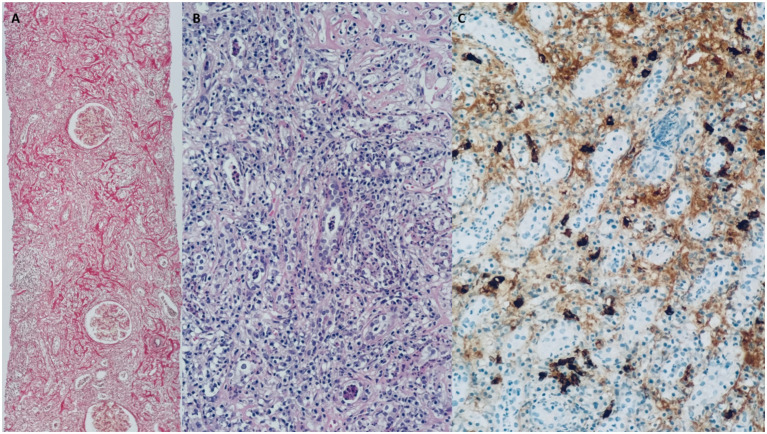
Histopathological findings consistent with IgG4-related disease. **(A)** Sirius Red Elastica staining; 100x. Storiform fibrosis of the parenchyma with mixed inflammatory cells. **(B)** Hematoxylin and eosin; 200x. Mixed inflammatory infiltrate with several plasma cells. Neutrophils localized within tubular lumina, epithelium and interstitium. **(C)** Immunohistochemistry, IgG4; 400x. Numerous IgG4 positive plasma cells.

In conjunction with the imaging pattern and absence of histologic malignancy, the lesions were interpreted as ICI-induced renal IgG4-RD rather than metastatic progression.

Given the negative ANCA serology and the absence of granulomatous inflammation on biopsy, ANCA-associated vasculitis was considered unlikely. ANA and ENA antibodies were also negative, and the patient did not meet the classification criteria for systemic lupus erythematosus or Sjögren’s disease. Allergic tubulointerstitial nephritis was considered unlikely, as the patient had not received any new medications or concomitant therapies other than the oncological treatment, with no apparent exposure to typical drug-related triggers (D).

Given concerns that systemic glucocorticoids could attenuate antitumor immune responses, the case was discussed in a multidisciplinary team involving oncology, rheumatology, nephrology, pathology, and immunology specialists. Rituximab was selected as steroid-sparing first-line therapy and administered intravenously at a dose of 1000 mg on days 1 and 15 in July 2024.

Follow-up CT performed in September 2024 demonstrated partial regression of pulmonary metastatic nodules and stabilization with interval improvement of renal lesions (**Figure 4**). No further renal deterioration occurred, and the patient maintained excellent clinical performance status (PS 0) throughout follow-up.

**Figure 4:**
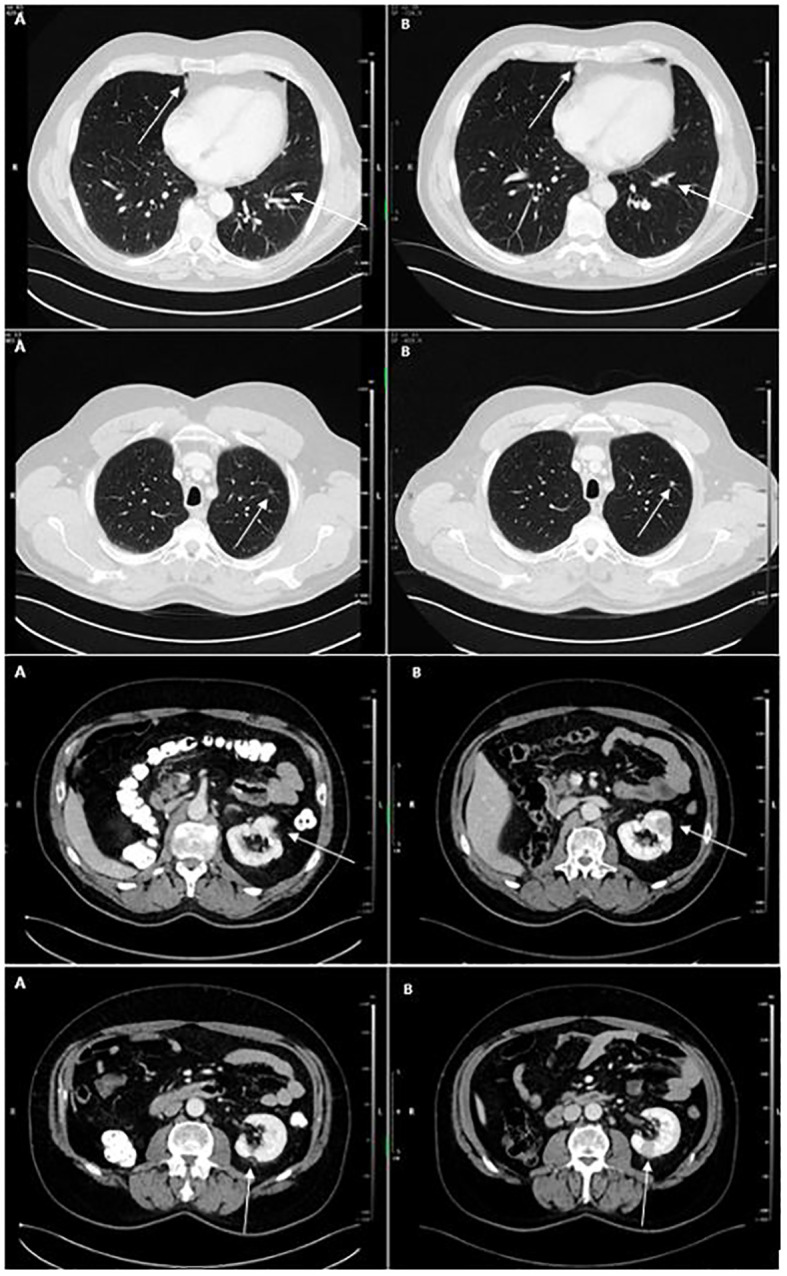
Regression of lung and kidney lesions March 2025 **(A)** in comparison with lesions March 2024 **(B)** - arrows.

## Discussion

This report expands the emerging spectrum of rheumatic and fibroinflammatory irAEs associated with dual immune checkpoint blockade. While musculoskeletal complaints such as arthralgia, inflammatory arthritis, myalgia, sicca syndrome, and vasculitic syndromes are increasingly recognized during ICI therapy, IgG4-RD remains among the rarest reported manifestations (4–10).

The mechanistic relationship between ICIs and IgG4-RD is biologically plausible but remains insufficiently explored. Classical IgG4-RD is driven by a complex interaction between activated CD4+ cytotoxic T cells, T-follicular helper (Tfh) cells, plasmablast expansion, and profibrotic cytokine production. PD-1 and CTLA-4 signaling pathways are central regulators of peripheral T-cell tolerance and B-cell help (7, 8). Their pharmacologic blockade may therefore facilitate exaggerated Tfh-cell activation, loss of regulatory restraint, and enhanced B-cell class switching toward IgG4-producing plasmablasts, ultimately promoting fibroinflammatory tissue injury. This concept is supported by the observation that many ICI-induced autoimmune toxicities phenotypically recapitulate classical systemic immune diseases but often with atypical organ distribution or accelerated onset (13).

A major clinical challenge is that ICI-induced IgG4-RD may closely mimic cancer progression. In our patient, newly developed renal mass lesions in a solitary kidney were initially highly suspicious for metastatic recurrence. Without tissue confirmation, this could have prompted major changes in oncologic strategy or unnecessary escalation of anticancer therapy. Histopathology therefore remains essential whenever new organ lesions arise in atypical clinical contexts during ICI treatment.

Another important aspect concerns treatment selection. High-dose glucocorticoids remain standard first-line therapy for most severe irAEs; however, prolonged corticosteroid exposure may theoretically blunt antitumor T-cell surveillance and has been associated in some studies with inferior oncologic outcomes when introduced early or at high doses. Their prolonged use may be associated with clinically relevant disadvantages. In addition to well-established complications, including increased susceptibility to infections, metabolic disturbances, osteoporosis, and cardiovascular morbidity, systemic corticosteroids may theoretically counteract the antitumor immune response induced by immune checkpoint blockade. Several retrospective studies have suggested that baseline or early corticosteroid exposure, particularly when administered for cancer-related indications or at higher doses, may be associated with reduced efficacy of ICI therapy and inferior clinical outcomes (14). Although these findings may be influenced by confounding factors, they highlight the importance of minimizing unnecessary prolonged immunosuppression whenever clinically feasible.

Therefore, steroid-sparing approaches using targeted immunomodulatory therapies may represent an attractive strategy for selected irAEs, particularly when the underlying immune-mediated process is driven by specific pathogenic pathways. **(E)** Because B-cell/plasmablast expansion is central to IgG4-RD pathobiology, rituximab represents a rational targeted alternative (10). Indeed, rituximab is highly effective in conventional IgG4-RD and may allow disease control while minimizing broad T-cell immunosuppression. In our patient, B-cell depletion was associated with stabilization/regression of fibroinflammatory renal lesions together with preserved oncologic control, supporting this steroid-sparing strategy.

To our knowledge, only a handful of similar cases of ICI-associated IgG4-RD have been published, including autoimmune pancreatitis, hypophysitis, and retroperitoneal fibrosis after nivolumab-based regimens (9, 10). Our report further extends this spectrum by documenting biopsy-proven renal pseudotumoral involvement successfully managed with rituximab.

## Conclusion

IgG4-related disease should be considered in the differential diagnosis of newly emerging mass-like lesions during or after immune checkpoint inhibitor therapy. Because its radiologic presentation may closely resemble metastatic progression, histologic verification is crucial. This case further suggests that rituximab may provide an effective steroid-sparing therapeutic option when preservation of antitumor immunity is clinically desirable.

## Data Availability

The original contributions presented in the study are included in the article/supplementary material, further inquiries can be directed to the corresponding author/s.
